# Non suicidal self injury and suicidal behavior among adolescents: co-occurrence and associated risk factors

**DOI:** 10.1186/s12888-022-03763-z

**Published:** 2022-02-09

**Authors:** Anju Poudel, Anjana Lamichhane, Kamala Rana Magar, Gopal Prasad Khanal

**Affiliations:** 1 Tribhuvan University, Institute of Medicine, Pokhara Nursing Campus, Pokhara, Nepal; 2 Satvik Nepal, Pokhara, Nepal; 3 Health Directorate, Pokhara, Gandaki Province Nepal; 4 Pokhara Academy of Health Sciences, Pokhara, Nepal

**Keywords:** Non suicidal self injury, NSSI, Suicidal behavior, Suicidal attempt, Suicidal ideation

## Abstract

**Background:**

Non-suicidal self-injury (NSSI) and suicidal behavior (SB) are the major public health problems in adolescents. Despite the increased focus on these phenomena, there exist no reliable data in Nepal. This study aimed to determine the prevalence of NSSI and SB among adolescents. Furthermore the study identified the relationship between these two behaviors and assessed demographic, behavioral, and psychological risk factors of NSSI and SB in Nepalese adolescents in a representative sample of the general population.

**Methods:**

The study was conducted among 730 adolescents studying in grade 9 to 12 of public and private schools of Pokhara Metropolitan city, Nepal. Data were collected through self administered standard tools- Functional Assessment of Self Mutilation (FASM) tool, Suicidal Behaviors Questionnaire-Revised (SBQ-R), Rosenberg self-esteem scale (RSES) and Beck Depression Inventory (BDI). Descriptive statistical measures such as frequency, percentage, mean, standard deviation, range were used to assess demographic characteristics and adolescent’s behavior regarding NSSI and suicide. For inferential analysis chi-square and one way ANOVA test was used. Furthermore, to determine the predictors of NSSI and SB, multiple logistic regression analysis was used.

**Results:**

Regarding behavioral characteristics, nearly half of the sample 327 (44.8%) reported a history of NSSI in past 1 year. Furthermore, 25.8% (*n* = 188) of the overall sample engaged in minor NSSI only and 3.42% (*n* = 25) engaged in at least one act of moderate/severe NSSI. The mean number of type of NSSI performed was 2.63 ± 1.71. The most common type of NSSI method used were picking at wound (27.3%), biting self (20.3%), pulling hair out (11.8%), cutting self (11.1%). Boys (52.6%) were more likely to be engaged in NSSI than girls (47.4%) (*χ*^2^  =  10.298, *p*  =  0.002). Furthermore, among 730 adolescents who completed the SBQ-R questionnaire, 131 (17.9%) had suicidal behaviors (SB) (as defined by SBQ-R a total score ≥ 7). Regarding sex differences female were significantly higher in life time prevalence of suicidal behavior than male (*χ*^2^ = 30.26, *p* = 0.001). Simple Chi-square tests indicated that NSSI was significantly associated with SB (*χ*^2^ = 58.16, *P* < .001). Logistic regressions identified the four significant predictors of NSSI behavior: male, low-level of self-esteem (SE), moderate to severe form of depression and SB. Similarly, significant predictors of SB were: female, low-level of SE, moderate to severe form of depression and NSSI behavior.

**Conclusion:**

The prevalence of both NSSI and SB is high in adolescents. Despite the differences between NSSI and SB a significant number of adolescents reported a history of both behaviors. Lower level of SE and moderate to severe depression were the significant predictors of both NSSI and SB. Furthermore, male and adolescents with the history of SB were at risk of NSSI behavior whereas female sex and adolescents with the history of NSSI were at risk of SB.

## Background

Non suicidal self injury (NSSI) and suicidal behavior (SB) are the common self harm behaviors among the adolescents and young adults. NSSI refers to an act involving the direct and deliberate destruction of one’s body tissue using methods that are not socially or culturally sanctioned and without the intent to kill oneself [1]. The most common examples among adolescents includes the behaviors such as scratching, banging, burning, cutting, self hitting body parts and interfering with wound healing [2–4]. On the other hand suicide refers to self injurious behaviors with the intent to end one’s life [5]. Therefore, it is important to differentiate NSSI from suicidal behavior based on recent literatures consensus on “with” and “without” intent to die [6, 7]. Furthermore, suicide related thought and behaviors are classified into: suicide ideation, suicidal plan, suicidal threat and suicide attempt. *Suicide ideation* is defined as considering or thinking about engaging in behavior intended to end one’s life; *suicide plan* refers to the proposed method of self-injury through which one intends to die; *suicide threat *refers to any verbal or non-verbal action intended to communicate that suicidal behavior might occur in near future and on the other hand *suicide attempt* refers to engagement in potentially self-injurious behavior in which there is at least some intent to die [6, 8].

A substantial body of research related to NSSI and SB has been conducted in developed countries [2, 9, 10], highlighting the need of such studies. But it has been neglected entirely in low- and middle-income countries [11] and only few studies from Nepal has acknowledged on this area [12–14]. Most of the previous studies concentrated among clinical populations suggested high prevalence of NSSI among psychiatric patients and its association with various psychiatric problems [15, 16] whereas the recent studies highlights a high prevalence of NSSI in nonclinical populations specially community adolescents [17, 18]. A systematic review including 52 international studies analyzed the prevalence of NSSI in adolescents and reported a rate of 18% lifetime prevalence of NSSI in community sample [19]. A higher rate i.e. 34% of adolescents engaging in NSSI at some point in their lives was observed in a study conducted in US [20] whereas other study reported that 29.5% of adolescents were engaged in NSSI, 7.1 to 8.9% had elevated levels of suicidal ideation (SI), and 3.3% of adolescents had suicidal attempt (SA) in the past year [21]. Donath et al. [9] also reported a lifetime prevalence rate of SI 36.6%, SA 7.6% and 1 year prevalence of direct self injurious behavior 17.8% in a representative sample of German students. In a pooled analysis of the Global School-based Student Health Survey (GSHS) across 59 low-income and middle-income countries, the prevalence of SI and SA was found to be 16.9 and 17.0% respectively, highest in the African region and lowest in the Southeast Asia region [22]. GSHS in Nepal reported the prevalence of SI 13.6% and SA 10.3% [13].

NSSI has been associated with a broad array of self-reported functions, including emotion-regulation, self-punishment or communication of distress [2, 23–26]. Therefore, this behavior is distinguished from SA by the absence of a conscious desire to die. Although NSSI is distinct from SA, some studies indicate that 50–75% of those individuals with a history of NSSI also have made SA [27, 28]. Numbers of other studies have also shown that NSSI is associated with SB and it is a significant predictor of subsequent NSSI and SA [10, 21, 29–34]. Among 2131 middle school Chinese adolescents, the lifetime prevalence of NSSI and SA was 23.2 and 3.2% respectively while the co-occurrence of these two behaviors was reported by 2.3% [33]. Furthermore, it has been suggested that high frequencies of NSSI incidents were associated with a significant increase in risk of SI and SA. Each additional unit increase in reported NSSI was associated with a seven-fold increased likelihood of future SA [21]. Anestis et al. [34] also found that 33.6% of individuals with prior history of NSSI had ever attempted suicide compared to only 2.5% of individuals with no prior history of NSSI. The frequent observations of the coexistence of NSSI and SB highlight the essence of assessment of the nature of the link between these two types of behavior. In addition, it is important to underline the factors that differentiate adolescents engaging in NSSI from SA since not all the adolescents who engage in NSSI attempt suicide, and not all the individuals who attempt suicide engage in NSSI.

Despite sound knowledge on human psychology and continuous advancement in neurosciences, suicidal behaviors are difficult to understand [35]. Scientific evidences highlight the role of genetics, various stressors, the hypothalamic-pituitary-adrenal stress-response system, the involvement of the monoaminergic neurotransmitter systems, the lipid profile, neuro-immunological biomarkers, the brain-derived neurotrophic factor and other neuromodulators on the complex link between suicidal behavior and depression [36]. Notably, suicidal behavior has been implicated as a co-morbidity of several neuropsychiatric disorders. Recent articles, reporting individual cases have also illustrated the link between depression and suicidal behaviors [37, 38]. Moreover, studies have shown clear association between depressive symptoms and NSSI in adolescents and young adults, both in community [3, 39] and clinical samples [27, 39]. Depression, anxiety, NSSI history, female gender and younger age are often reported as the predictor of NSSI [40, 41]. Similarly, depressive disorders and NSSI history are the strongest risk factor for SI and SA [30, 41–43]. The adolescents with a history of both attempted suicide and NSSI generally experience more psychological symptoms than adolescents who have engaged in only one type of self-injury [44, 45]. SA and SA plus NSSI group are significantly more likely to be diagnosed with major depressive disorder than only NSSI group [39, 46]. Similarly, Brausch et al. also reported that NSSI group have fewer depressive symptoms, lower SI, and greater self-esteem (SE) than the group that also had attempted suicide [47].

NSSI and SB continue to be absolutely devastating problems among children and adolescents. It has been recognized as a public health problem with high burden in low and middle income countries. Despite the increased interest and pursuit of research into NSSI and SB, there exists no reliable estimate of the prevalence of NSSI and SB in the general Nepalese adolescents. Therefore, the objectives of the current study are

- to assess the prevalence of NSSI and suicidal behavior among adolescents.
- to identify the characteristics of NSSI and suicidal behavior among adolescents
- to assess association between NSSI and suicidal behavior
- to identify the predictors of NSSI and suicidal behavior

The study also sought to fulfill the gap in literature related to NSSI and suicide from developing country like Nepal.

## Methods

A cross sectional survey was conducted at selected public and private secondary level schools of Pokhara Metropolitan city, Nepal.

### Participants and procedure

Two stage cluster sampling technique was applied to select representative sample from study population that comprised of 9 to 12 class students. At first, list of the public and private schools and number of students of each class were obtained from District Education Office, Kaski. Number of students in each class ranged from 30 to 50. In initial stage of sampling strategy, to meet the sample size, 5 public and 5 private schools were chosen from among the total secondary level schools (9 to 12 classes), using simple random sampling technique (lottery method). In second stage, intact classrooms from each of chosen school were selected to participate in the study and each student of selected classroom was eligible to participate in the study. The inclusion criteria for the participation in the study were that the students should of grade 9 to12 and willing to participate in the study.

Sample size was calculated by using the formula n = z^2^pq/d^2.^ According to the study conducted in Kathmandu, Nepal at tertiary care centers, the overall prevalence of lifetime self-harm behavior among secondary level students is 55.6% [14]. So, using this prevalence with an allowable error of 5% at a confidence interval of 95%, the sample size was estimated to be 380. To minimize the design effect, sample was multiplied by 1.5 as cluster sampling technique was adopted. Assuming a non-response rate of 20%, calculated sample size was 684**.**

A total of 748 questionnaires were distributed to students of selected classrooms and all of them submitted the questionnaires to the researcher (student response rate = 100%). Of them, 18 questionnaires missed the important information or were not readable, thus excluded from the study. Finally 730 questionnaires (382 from public schools and 348 from private schools) were included in the analysis.

### Measures

A semi structured self administered questionnaire was used and it consisted of different parts.

#### Part I

Socio-demographic questions included participants age, sex, type of school (Public, Private).

#### Part II

NSSI was assessed through Functional Assessment of Self Mutilation (FASM) tool [48]. FASM consists of a checklist of 12 types of NSSI, denoted as ‘minor NSSI’: hitting self, pulling hair, biting self, inserting objects under nails or skin, picking at a wound, and picking areas to draw blood; ‘moderate/severe NSSI’: cutting/carving, burning, self-tattooing, scraping, and erasing (i.e. using an eraser to rub skin to the point of burning and bleeding) skin [2]. The questionnaire also consists of NSSI additional information questions, and a checklist of potential functions of NSSI. However in this study NSSI functions were not assessed. This tool has been used in several studies [2, 24, 27, 49]. The FASM has demonstrated acceptable psychometric properties within adolescent samples, yielding adequate internal consistency ranging from 0.62 to 0.85 for each subscale of functions of NSSI [25].

#### Part III

SB was assessed by using Suicidal Behaviors Questionnaire-Revised (SBQ-R). SBQ-R consisted of four items, each assesses different dimension of suicidality: lifetime suicide ideation and / or suicide attempt, frequency of SI over the past 12 months, threats of SA, and self-reported likelihood of SB in the future. Items are scored using a Likert-scale, with between 5 and 7 response choices per item, and are summed for a total score (range 3–18) [50]. Higher score indicates higher levels of suicidality. Furthermore, the cut-off score between suicidal behavior (SB) and non-suicidal behavior for the undergraduate sample is identified as a score of 7 (i.e., a score of 7 or above will be classified as suicidal). The internal consistency coefficients of the SBQ-R were 0.76–0.88 in the Osman et al. study [50].

#### Part IV

Depression was assessed by using Nepali version of the 21- item Beck Depression Inventory (BDI) [51]. The BDI is a commonly used self- measure of depressive severity that includes 21 questions about various symptoms of depression. Each answers being scored on a scale value of 0 to 3. Total scores can range from 0 to 63, with higher scores indicating more depression. The standardized cutoff point is: 0–13: minimal depression, 14–19: mild depression, 20–28: moderate depression, 29–63: severe depression. The Nepali version of BDI has excellent psychometric properties (Cronbach’s alpha = 0.90 and 2 week test-retest reliability = 0.84) [51, 52].

#### Part V

SE was assessed by Rosenberg Self-esteem scale (RSES). It is a 10-item measure that assesses an individual’s overall evaluation of his or her worth or value [53]. Globally, RSES is the most widely used scale to measure SE among adolescents and has been shown to have good reliability. The items are rated using a 4-point likert scale (0 = strongly disagree; 3 = strongly agree). The scoring is obtained by doing the sum of scores according to the ratings assigned to all the items after reverse scoring the negatively worded items and score ranges from 0 to 30. The score less than 15 indicate a problematic low self esteem. The internal consistency reliability of tool ranges from 0.85 to 0.88 for college samples [53].

Apart from BDI which was available in Nepali language, other tools were translated into Nepali language using standard guideline [54].

### Data collection procedure

Data was collected on August 2019 after getting ethical approval from Ethical Review Board of Nepal Health Research Council (Ref. No 3051). Informed verbal consent was obtained from the school authority after explaining the purpose of the study. One day before data collection the parents/care giver of all selected participants were informed about the study objectives, nature of the survey, participants rights regarding voluntariness and withdrawn form study through a letter sent to home with their child. Parent’s permission to include their child in the study was ensured though signature of the parents in the informed consent form. Furthermore, verbal and written informed consent was obtained from the subjects prior to data collection. One-class period (approx. 45 min) was provided to fill in the questionnaire. Considering the sensitivity of the issue, the school authority was requested not to be present in the class during the filling in of the questionnaire. However, researcher was available to make clear of any queries if present. Students were assured that the information they provided would remain confidential and thus were encouraged to be truthful in their responses. They were informed that their participation was completely voluntary and they could quit at any time if they feel uneasy to give answer.

### Data analysis

Statistical Package for Social Sciences (Version 20; IBM Corporation, Armonk, NY, USA) was used for data analysis. Normality was verified using Shapiro–Wilk test, and the data were found to be normally distributed (*p* > 0.05). For the detail analysis of the item 1 of SBQ-R question, the sub-categories: suicidal ideation, suicidal plan and suicidal attempt were entered separately. Furthermore, based on the SBQ-R cut of point (i.e. Suicidal behavior (SB): total score ≥ 7), adolescents were further categorized into No-NSSI +No SB group, NSSI only group, SB only group and NSSI+SB group. The data was analyzed by using descriptive statistics such as frequency, percentage, mean and standard deviation. For inferential analysis chi-square test was used for categorical variables and ANOVA test for continuous variable. Furthermore, multiple logistic regression was done to identify the predictors of NSSI and SB.

## Results

Among 730 adolescents participated in the study 382 (52.3%) were from public and 348 (47.7%) were from private schools. Almost equal percent, 336 (46%) were male and 394 (54%) were female. The mean age of the participants was 15.85 ± 1.31, ranging from 12 to 19 years. They were categorized into early adolescents (10–14 years) 107 (14.7%) and late adolescents (15–19 years) 623 (85.3%).

### NSSI behavior of adolescents

Regarding behavioral characteristics, nearly half of the sample 327 (44.8%) reported a history of NSSI in past 1 year. Furthermore, 25.8% (*n* = 188) of the overall sample engaged in minor NSSI only and 3.42% (*n* = 25) engaged in at least one act of moderate/severe NSSI. Twenty seven percent of self-injurers (*n* = 87) reported engaging in only one type of NSSI in the past year, while 66% (*n* = 217) of self-injurers endorsed engaging in two to five different types of NSSI, and 7% (*n* = 23) endorsed six or more different types of NSSI. The mean number of type of NSSI performed was 2.63 ± 1.71 (median = 2.0, mode = 2.0, range 1–10). Among those adolescents who engage in NSSI in the past year, the average number of incident was 9.12 (SD = 9.95, median = 6·0, mode = 1·0, range = 1–102) (Table 1).

**Table 1:** Behavioral characteristics of adolescents

				*n* = 730
Variables	Category	Number	%	M ± SD (R = Range)
NSSI behavior	No NSSI	403	55.2	
	Minor NSSI only	188	25.8	
	Moderate /Severe NSSI only	25	3.42	
	Both type of NSSI	114	15.6	
Method of NSSI Performed(*n* = 327)	Only one type	87	27.0	2.63 ± 1.71 (R = 1–10)
	Two to five type	217	66.0	
	Six or more type	23	7.0	
Frequency of NSSI performed (*n* = 327)				9.12 ± 9.95 (Range = 1–102)
Suicidal Behavior	No Suicidal Behavior	599	82.1	4.44 ± 2.30 (R = 3–13)
	Suicidal Behavior	131	17.9	
Depression	Minimal range	440	60.3	13.36 ± 10.31 (R = 0–53)
	Mild depression	120	16.4	
	Moderate depression	95	13.0	
	Severe depression	75	10.3	
Self-esteem	Low self-esteem	75	10.3	24.53 ± 7.24 (R = 0–30)
	High self-esteem	655	89.7	

The type of NSSI method used were picking at wound (27.3%), biting self (20.3%), pulling hair out (11.8%), cutting oneself (11.1%), tattoing (9.6%), hitting self on purpose (9.2%), inserting objects under nails and skin (8.1%), picking areas of own body to the point of drawing blood (7.5%), erased skin (4.2%), burning skin (3.4%) and scraping own skin (2.3%). Regarding frequency of behavior, the most frequently reported forms of NSSI were also picking at the wound, biting self, pulling hair out. Of moderate to severe NSSI giving self tattoo and cutting on skin was common.

Boys (52.6%) were more likely to be engaged in NSSI than girls (47.4%) (*χ*^2^  =  10.298, *p*  =  0.002). Similarly, adolescents studying in private schools (53.2%) reported more NSSI behavior than adolescents of public schools (37.2%)(*χ*^2^  =  10.298, *p*  =  0.002). None of the adolescents reported engaging in NSSI while taking drugs or alcohol, and the majority of them (75%) reported little or no pain, with the remaining 4% reported severe pain. The mean age of first NSSI incident was 13.57 ± 1.71 years and ranged from 10 to 17 years. Seventeen percent (*n = 56*) of the self-injurers reported receiving medical treatment as a result of their self-injurious behavior.

### Suicidal behavior of adolescents

Among 730 adolescents who completed the SBQ-R questionnaire, 131 (17.9%) had SB (as defined by SBQ-R, a total score ≥ 7). Similarly, 120 (16.4%) had mild depression, 95 (13.0%) had moderate depression and 75 (10.3%) had severe depression. Regarding SE 75 (10.3%) had low SE and 655 (89.7%) had high SE (Table 1).

Furthermore, the percentage of participants who reported past SI, past suicidal plans (SP) and past SA were also calculated based on Item 1 of the SBQ-R. The lifetime prevalence of SI, SP an and SA was, 76(10.4%), 58(7.9%) and 33(4.5%) respectively. Similarly, regarding frequency of SI in the past 1 year, 76(10.4%) had only once, 50 (6.8%) had twice, 16(2.2%) had 3 to 4 times and 17 (2.3%) had 5 or more times SI in a year. One hundred sixty three (22.3%) adolescents had threat of SA or they told to other people as they were going to commit suicide. Out of those, 103 (63.2%) adolescents told once and 60 (36.8%) adolescents told more than once to others. The likelihood of SB in future was reported only by 3 (0.4%) adolescents (Table 2).

**Table 2:** Magnitude of Suicidal Behavior among adolescents

		*n* = 730	
Variables	Category	n	Percent
**Life time suicidal ideation, intent and/or attempts**	Never	563	77.1
	Suicidal ideation	76	10.4
	Suicidal Plan	58	7.9
	Suicide attempts	33	4.5
**Frequency of suicidal ideation in the** **past 1 year**	Never	571	78.2
	Once	76	10.4
	Twice	50	6.8
	3–4 times	16	2.2
	Always (≥ 5 times)	17	2.3
**Suicidal threats**	Never	567	77.6
	Once	103	14.1
	Twice and more	60	8.2
**Likely hood of suicide in the future**	Never	564	77.3
	No chance at all	94	12.9
	Rather unlikely	63	8.6
	Unlikely	6	0.8
	Likely	3	0.4

Regarding sex differences female were significantly higher in life time prevalence of SB than male (***χ***^**2**^ = 30.26, *p* = 0.001). No significant differences were noted between private and public schools in SB.

### Association between NSSI and suicidal behavior

Simple Chi-square tests indicated that NSSI was significantly associated with SB (***χ***^**2**^ = 58.16, *P* < .001). Of those who had SB, 74.8% had also engaged in NSSI and of those who reported NSSI, 30.0% also had the history of SB. Separate analysis of the elements of SB revealed that lifetime prevalence was significantly higher among NSSI adolescents than No-NSSI adolescents for suicidal ideation (17.4% vs 4.7%; OR: 4.2 [95%CI, 2.4–7.3]; *P* < .001) and plan (8.6% vs 7.4%; OR, 1.1 [95%CI, 0.6–1.9]; *P* < .001) and attempt (8.0% vs 1.7%); OR, 4.8 [95%CI, 2.0–11.4]; *P* < .001) (Table 3).

**Table 3:** Association between NSSI and Suicidal Behavior

				*n* = 730
NSSI behavior	Suicidal Behavior			
	No Suicidal Thought	Suicidal Ideation	Suicidal Plan	Suicidal Attempt
**No- NSSI**	347 (86.1%)	19 (4.7%)	30 (7.4%)	7 (1.7%)
**NSSI**	216 (66.1%)	57 (17.4%)	28 (8.6%)	26 (8.0%)
**OR (95%CI)**	3.1 (2.2–4.5)	4.2 (2.4–7.3)	1.1 (0.6–1.9)	4.8 (2.0–11.4)
*P*-value	<.001	<.001	<.001	<.001

### Group differences

Based on the adolescent’s NSSI and SB they were categorized into different groups. The No-NSSI +No SB group included 370 (50.6%) adolescents, the NSSI only group had 229 (31.3%) adolescents, SB only group had 33 (4.5%) adolescents and NSSI + SB group had 98 (13.4%) adolescents. Significant differences emerged between groups in terms of all demographic and psychological characteristics. SB only group were predominantly female and from public school compared to three other groups. Furthermore, all the four groups were mostly late age adolescents (15–19 years), and the difference was significant (Table 4).

**Table 4:** Characteristics of NSSI and SA adolescents

**Dichotomous Variable**	**No-NSSI + No SB**		**NSSI only**		**SB Only**		**NSSI + SB**		**Chi-square test (χ2)**	***p*-value**
	***n*** **= 370**		***n*** **= 229**		***N*** **= 33**		***n*** **= 98**			
	**n**	**%**	**n**	**%**	**n**	**%**	**n**	**%**		
Female	213	57.6	94	41.0	26	78.8	61	62.2	28.20	<.001*
Public School	217	58.6	93	40.6	23	69.7	49	50.0	22.73	<.001*
Late aged adolescent	300	81.1	208	90.8	30	90.9	85	86.7	11.85	.008*
**Continuous Variable**	**Mean**	**(SD)**	**Mean**	**(SD)**	**Mean**	**(SD)**	**Mean**	**(SD)**	**ANOVA test (*F*)**	***p*-value**
Depression	10.08	8.59	13.57	9.11	15.54	7.64	24.52	11.61	64.59	<.001*
Self-esteem	26.24	5.76	23.79	7.31	23.36	9.16	20.20	9.07	21.25	<.001*

*NSSI* Non-suicidal self injury, *SB* Suicidal behavior, * *p*-value significance at ≤ 0.05 level

In addition, data were analyzed using a one- way ANOVA with self-harm group as independent variable and depression and self-esteem as dependent variable. The NSSI+SB group were significantly more depressed and had lower level of self-esteem than NSSI only, SB only and No-NSSI +No SB groups (Table 4).

### Logistic regression to identify the predictors of NSSI and suicidal behaviors

Logistic regressions identified the four significant predictors of NSSI behavior: sex, SE, depression and SB. Dependent variable, NSSI behavior had two categories: No NSSI and NSSI adolescents. Female adolescents and those with higher SE were less likely to perform NSSI behavior. Similarly, compared to those who do not have suicidal thought, adolescents who have SI were 4.6 times, and those who had attempted suicide were 4.2 times higher risk of NSSI behavior. Furthermore, adolescents who had moderate to severe depression were 2.6 times higher risk of NSSI behavior as compared to minimal to mildly depressed adolescents (Table 5).

**Table 5:** Predictors of NSSI behavior

Predictor variable	B	SE	Odd ratio	***P*** value	95.0% CI for odds ratio
**Age**					
**Early adolescents (Reference)**					
**Late adolescents**	0.347	0.241	1.415	0.150	0.882 to 2.270
**Sex**					
**Male (reference)**					
**Female**	−0.858	0.172	0.424	**< 0.001**	0.302 to 0.594
**Suicidal behavior**					
**No Suicidal Thought (reference)**			4.648	**< 0.001**	2.608 to 8.282
**Suicidal Ideation**	1.536	0.295	1.209	0.542	0.657 to 2.224
**Suicidal Plan**	0.190	0.311	4.236	**0.002**	1.517 to10.460
**Suicidal Attempt**	1.444	0.461			
**Depression**					
**Minimal to Mild depression (reference)**					
**Moderate to Severe depression**	0.976	0.212	2.655	**< 0.001**	1.752 to 4.022
**Self-esteem**					
**Low self-esteem (reference**					
**High self-esteem**	−0.747	0.290	0.474	**0.010**	0.268 to 0.837

To identify the predictors of SB, age, sex, self-esteem, depression and NSSI behavior was entered as independent variables. Based on SBQ-R cut off point, SB was defined into two group: No SB and SB. Sex, SE, depression and NSSI behavior were the independent predictors of SB. Female adolescents compared to male adolescents were 1.7 times at risk of SB. Similarly, compared to those adolescents who do not perform NSSI, adolescents who perform only minor type of NSSI were 2.4 times, moderate to severe type of NSSI performing adolescents 8.5 times and those who perform both type of NSSI were 5.3 times higher risk of SB. Furthermore, adolescents with higher SE were less likely to exhibit SB than adolescents having lower SE and adolescents who had moderate to severe depression were 3.8 times higher risk of SB as compared to minimal to mildly depressed adolescents (Table 6).

**Table 6:** Predictors of Suicidal behavior

					*n* = 730
Predictor variable	B	SE	Odd ratio	***P*** value	95.0% CI for odds ratio
**Age**					
Early adolescents (Reference)					0.492 to 1.791
Late adolescents	−0.06	0.32	0.939	0.849	
**Sex**					
Male (reference)					
Female	0.55	0.237	1.733	**0.020**	1.090 to 2.757
**NSSI behavior**					
No NSSI (reference)					
Minor NSSI	0.883	0.274	2.418	**0.001**	1.414 to 4.134
Moderate to Severe NSSI	2.148	0.485	8.567	**< 0.001**	3.310 to 22.170
Both NSSI (Minor+Moderate to Severe NSSI)	1.672	0.294	5.321	**< 0.001**	2.993 to 9.459
**Depression**					
Minimal to Mild depression (reference)					
Moderate to Severe depression	1.356	0.234	3.882	**< 0.001**	2.452 to 6.147
**Self-esteem**					
Low self-esteem (reference)					
High self-esteem	−0.821	0.304	0.440	**0.007**	0.242 to 0.798

## Discussion

By definition, both NSSI and SA involve intentional harm to oneself but, unlike suicide attempts, NSSI does not involve intent to die. NSSI is a common but often hidden behavior, especially among adolescents. Nearly half of the sample 44.8% reported a history of NSSI in past 1 year. This proportion is fairly consistent with other studies [2, 55]. A previous study from Nepal also found higher rate of self harm behavior among adolescents with lifetime prevalence of 55.6% [14]. Similarly, a study with similar type of sample from US also reported that approximately 34% of the adolescents were engaged in some form of NSSI at some point in their lives, with 16% of the overall sample reporting evidence of repetitive NSSI in the past 6 months [20]. However, the present study prevalence rate is generally higher than earlier studies of adolescent community samples [9, 17, 18, 41, 47]. The difference in prevalence rates might be due to differences in the cultural background of the sample, as well as use of different tools to assess self injuring method.

Furthermore, 66% of self-injurers endorsed engaging in two to five different types of NSSI, and 7% endorsed six or more different types of NSSI which is almost similar to the study by Lloyd-Richardson et al. [2]. Boys were more likely to be engaged in NSSI than girls. However some other studies indicated higher prevalence in girls [21, 56] and some Nepalese and Chinese adolescents did not reveal significant gender differences in rates of self-harm behavior [14, 17]. The most frequently reported forms of NSSI were picking at the wound, biting self and pulling hair out. Self tattooing and cutting on skin were common form of moderate to severe type of NSSI. Study conducted by Giri in Nepal also found scratching the skin, tattooing, preventing wounds from healing and cutting skin as the commonly chosen method of NSSI [14]. Chinese adolescents also reported hitting, pinching, pulling hair and biting, the frequently occurring NSSI behavior [17]. However some studies have suggested picking at the wound is clinically insignificant behavior [2] and biting self is a socially acceptable and normative behavior [57].

Suicide is one of the leading causes of death among adolescents around the world. In this study the life time prevalence of SB was 17.9%. In addition, the lifetime prevalence of SI, SP and SA were, 10.4, 7.9 and 4.5% respectively. Almost similar finding has been reported with the 1 year prevalence rate of 8.8 and 3.5% for SI and SA respectively in a study conducted in China [41]. However the prevalence rate found in our study is slightly lower than that reported by a global school health survey in Nepal which reported that nearly 14% of school students had SI while 10.3% had attempted suicide [13]. Global variation can be observed with prevalence of SI and SA, 4.9 and 6.7% in Bangladesh [58] and 11.6 and 11.3% in Bhutan [59] respectively. The heterogeneity across countries might be due to the effect of various risk factors associated with SB. Regarding sex differences female were significantly higher in life time prevalence of SB than male. It has been supported by many studies [13, 22, 33].

The present study found the significant association between NSSI and SB. NSSI was significantly associated with the increase odds of SI, SP and SA (OR: 4.2, 1.1 and 4.8 respectively). Of those who were engaged in NSSI, 8.0% of the adolescents had attempted suicide, which is significantly higher than that of people who were engaged in No- NSSI (1.7%). Furthermore, 13.4% of the overall sample reported co-occurrence of both NSSI and SB. Of those who had SB, 74.8% had also engaged in NSSI and of those who reported NSSI, 30% also had the history of SB. A high co-occurrence of NSSI and SA was observed among outpatient samples of adolescents where 37% of adolescents with a history of NSSI also reported having made at least one SA [29, 39]. A study by Nock et al. reported that 55–70% of adolescents with NSSI also had history of attempted suicide [29]. Moreover, a recent study found 7.7% lifetime prevalence of co-occurrence of NSSI and SB, among those with NSSI, 39.6% endorsed SB, and 66.3% of those with SB reported NSSI [60]. Consistent with the present study, Kiekens et al. [56] reported that NSSI was associated with increased odds of subsequent suicide ideation (OR = 2.8), plan (OR = 3.0), and attempt (OR = 5.5). These findings extend the previous work and provide evidence on co-occurrence and association between NSSI and SB [21, 31, 32, 41, 44, 56].

Despite the above evidence that NSSI and SB co-occur, these behaviors also differ in many ways in some characteristics [28, 29, 31, 33, 39, 44–47, 61]. In this study, significant differences were found between four groups (No NSSI and No SB, NSSI only, SB only, NSSI+SB) on age, sex, type of school, depression and self-esteem. SB only group and NSSI+SB group were predominantly female and from public school compared to NSSI only group. In addition, NSSI+SB group were significantly more depressed and had lower level of self-esteem than NSSI only, SB only and No-NSSI +No SB groups. Regarding gender, the finding has been contradicted by previous studies where the groups were predominated by female gender but the difference was not significant [39, 47] whereas significant difference was noted on a study conducted by Liang et al. [33]. Furthermore, in line with this study, the previous study found no self harm group reporting lower level of risk factors (hopelessness) and highest level of protective factor (self-esteem) compared to NSSI and NSSI+SA groups [47]. The other study also identified that adolescents in NSSI+SA group and SA only group were more likely to meet criteria for a major depressive disorder than NSSI only group [39]. The current study findings provide evidence that adolescents with both type of behavior are at greater risk of having sever form of psychological symptoms than adolescents with only one type of self harm behavior.

Furthermore, the study identified low self-esteem, moderate to severe form of depression, history of SI, history of SA and male gender as the significant predictors of NSSI, which differentiates adolescents with NSSI from No NSSI group. A study by Garisch et al. also reported that higher level of depression and lower self esteem are significantly associated with NSSI [55]. In addition, a recent review [40] highlighted depressive symptoms, suicidality and psychological distress as the important predictors of NSSI and our result further supports this finding. The same study [40] found female gender as the predictor of NSSI, however our study did not support this finding. NSSI theories proposed that people who engage in NSSI have particularly high levels of emotion dysregulation, and these feelings drive them to engage in NSSI as a way to improve their mood [62]. Moreover, prior history of suicidal thought and behavior has been found as the significant risk factor of NSSI [63, 64]. In addition, empirical evidence has demonstrated that NSSI is under-taken by some to alleviate negative emotions (such as low self-esteem) and may be used in times of difficulty in a person’s life [65]. Regarding gender in contrast to previous studies [30, 64] the current study revealed male as the significant predictor of NSSI.

Similarly, the lower level of self esteem, moderate to severe depression, female gender, minor and moderate to severe form of NSSI and engagement in both type of NSSI are the significant predictors of SB. These variables significantly differentiate the individuals with SB from without SB. Most of the previous studies suggest that NSSI is a strong risk factor for future SA [10, 11, 27, 30]. Nock et al. suggests that adolescents who use more varied methods to injure themselves are more likely to have a history of SA [27]. In addition, NSSI thought and higher frequencies of NSSI concurrently are associated with higher levels of suicide ideation and a history of SA [2, 3, 27, 28, 56]. Moderate to severe form of NSSI is associated with increased suicidal thoughts and behaviors compared to mild NSSI [2, 41] which is consistent with our study finding. Furthermore, among mental disorders, major depressive disorder is the strongest predictors of suicide and SB [42, 66]. In addition, a meta-analysis of the longitudinal studies identified low level of self-esteem as a significant risk factor of SA in adolescents and young adults [67].

The finding of this study has to be considered with reference to its limitations. The students who are more likely to have risk behaviors such as school not going students and drop out students were missed in the study which limits the generalizability of our findings. Furthermore, the study findings depend on the response of adolescents from self-administered questionnaire that might result in recall bias and unwillingness to provide their personnel information might have lead to either over reporting or under reporting of information. Moreover, there might be social desirability bias, since the existing stigma surrounding SB in culturally diversified Nepalese societies might have caused an underreporting of the conditions. In addition to this, the cross-sectional nature of the study prevents establishing a causal relationship between the variables. The lower level of self esteem and depression were either cause or consequences of NSSI and SB has not been identified. Thus, to solve this problem and identify the causal relationships, longitudinal and experimental studies need to be done.

## Conclusion

The prevalence of both NSSI and SB is high in adolescents. Despite the differences between NSSI and SB, a significant number of adolescents reported a history of both behaviors. Moreover, two of the strongest NSSI risk factors, lower level of self-esteem and depression were also the significant risk factors of SB. Furthermore, Male gender and history of SI and SA is the significant predictor of NSSI behavior. Similarly, female gender and adolescents with the history of NSSI behavior are at risk of SB. Thus the finding reinforces the importance of assessment of various risk behavior among the adolescents so that prevalence of NSSI and SB can be minimized.

### Implication of the study

Although there are plenty of study assessing NSSI behavior among adolescents from the USA, Canada, Australia and UK, data on adolescent NSSI are scarce from developing country like Nepal. Despite the above limitations, as per the researcher’s knowledge this is the first study to assess NSSI behavior among adolescents in Nepal. The study identified that large number of adolescents with risk behavior are in hidden form, that they need to be identified and appropriate intervention should be done. The finding provides valuable information to parents and teachers to assess the adolescent’s behavior and examine the psychological health periodically so that risk behavior like NSSI and suicide can be prevented by adopting appropriate preventive strategies. The study findings could be useful for policy makers in designing appropriate preventive strategies for suicide prevention programs. Furthermore, the significant association found between NSSI and SB; and higher level of depression and low level of self-esteem in NSSI+SB group emphasizes the potential importance of identifying and treating adolescents with a history of NSSI, and continuous assessment of adolescents’ mental health status. In addition, sex, self-esteem and depression were identified as the significant predictors of NSSI and suicidal behavior (which includes suicidal ideation, suicidal plan and suicidal attempt), that highlight the importance of consideration of socio-demographic characteristics and psychological factors on the occurrence of these negative outcomes. Understanding the reasons of NSSI is crucial for treatment; and future research needs to elaborate further on previous work. Furthermore, studies exploring the association of self-esteem with NSSI and SB among adolescents would be warranted as there is scare of literatures on this topic.

## Data Availability

The dataset supporting the conclusions of this study is included within the article. Regarding different tools used in this study, it is a copyright tool. The translated Nepali version of this tool are however available from the corresponding author upon request and with permission obtained from copyright owner of the tool.

## Abbreviations

NSSI: Non Suicidal Self Injury
FASM: Functional Assessment of Self Mutilation
SBQ-R: Suicidal Behaviors Questionnaire-Revised
BDI: Beck Depression Inventory
RSES: Rosenberg Self-Esteem Scale
SB: Suicidal Behaviour
SA: Suicidal Attempt
SP: Suicidal Plan
SI: Suicidal Ideation
SE: Self-esteem
GSHS: Global School-based Student Health Survey
